# Characterizing the progression from mild cognitive impairment to dementia: a network analysis of longitudinal clinical visits

**DOI:** 10.1186/s12911-024-02711-z

**Published:** 2024-10-18

**Authors:** Muskan Garg, Sara Hejazi, Sunyang Fu, Maria Vassilaki, Ronald C. Petersen, Jennifer St. Sauver, Sunghwan Sohn

**Affiliations:** 1 Department of Artificial Intelligence & Informatics, Mayo Clinic, 200 First St SW, Rochester, MN 55905 USA; 2 Department of Quantitative Health Sciences, Mayo Clinic, Rochester, MN USA; 3 University of Texas Health Science Center, Houston, TX USA; 4 Department of Neurology, Mayo Clinic, Rochester, MN USA; 5 College of Medicine, University of Florida, Gainesville, FL USA; 6 Department of Industrial Engineering and Management Systems, University of Central Florida, Orlando, FL USA

**Keywords:** Electronic health record, Mild cognitive impairment, Dementia, Progression of disease, Time varying

## Abstract

**Background:**

With the recent surge in the utilization of electronic health records for cognitive decline, the research community has turned its attention to conducting fine-grained analyses of dementia onset using advanced techniques. Previous works have mostly focused on machine learning-based prediction of dementia, lacking the analysis of dementia progression and its associations with risk factors over time. The black box nature of machine learning models has also raised concerns regarding their uncertainty and safety in decision making, particularly in sensitive domains like healthcare.

**Objective:**

We aimed to characterize the progression of health conditions, such as chronic diseases and neuropsychiatric symptoms, of the participants in Mayo Clinic Study of Aging (MCSA) from initial mild cognitive impairment (MCI) diagnosis to dementia onset through network analysis.

**Methods:**

We used the data from the MCSA, a prospective population-based cohort study of cognitive aging, and examined the changing association among variables (i.e., participants’ health conditions) from the first visit of MCI diagnosis to the visit of dementia onset using network analysis. The number of participants for this study are 97 with the number of visits ranging from 2 visits (30 months) to 7 visits (105 months). We identified the network communities among variables from three-fold collection of instances: (i) the first MCI diagnosis, (ii) progression to dementia, and (iii) dementia diagnosis. We determine the variables that play a significant role in the dementia onset, aiming to identify and prioritize specific variables that prominently contribute towards developing dementia. In addition, we explore the sex-specific impact of variables in relation to dementia, aiming to investigate potential differences in the influence of certain variables on dementia onset between males and females.

**Results:**

We found correlation among certain variables, such as neuropsychiatric symptoms and chronic conditions, throughout the progression from MCI to dementia. Our findings, based on patterns and changing variables within specific communities, reveal notable insights about the time-lapse before dementia sets in, and the significance of progression of correlated variables contributing towards dementia onset. We also observed more changes due to certain variables, such as cognitive and functional scores, in the network communities for the people who progressed to dementia compared to those who does not. Most changes for sex-specific analysis are observed in clinical dementia rating and functional activities questionnaire during MCI onset are followed by chronic diseases, and then by NPI-Q scores.

**Conclusions:**

Network analysis has shown promising potential to capture significant longitudinal changes in health conditions, spanning from the MCI diagnosis to dementia progression. It can serve as a valuable analytic approach for monitoring the health status of individuals in cognitive impairment assessment. Furthermore, our findings indicate a notable sex difference in the impact of specific health conditions on the progression of dementia.

## Introduction

According to the World Health Organization, there are currently 50 million individuals (about twice the population of Texas) living with dementia worldwide, and this number is expected to triple by 2050 [29]. In the United States, the prevalence of dementia is already significant, with over 7 million individuals (about twice the population of Oklahoma) aged 65 or older reported to have the condition in 2020. Moreover, if current demographic and health trends persist, it is predicted that the number of Americans with dementia will exceed 9 million by 2030 and 12 million by 2040 [38].

Dementia impacts the individual’s cognitive ability of making safer decisions, such as driving or managing finances and hence, its early detection may also help the families of patients living with dementia take appropriate steps to ensure the safety of their loved ones. Despite the presence of specific diagnostic criteria, accurate dementia diagnosis is challenging, particularly in the initial stages, resulting in mis-diagnosed or delayed diagnosis of dementia. Early signs of dementia may be present in patients’ evaluations, several years before actual diagnosis [17]. As there is currently no cure available for dementia, the early detection of dementia is critical to allow patients and families to plan, receive the available interventions and support, and take advantage of the limited treatment options available [1].

The clinical data of a population-based cohort offers an opportunity to study the progression from first MCI diagnosis to dementia by analyzing longitudinal clinical visit data. The progression characterization of dementia onset refers to how it develops over time, including the symptoms that arise and the rate at which they progress. For instance, the early signs of dementia are forgetfulness, difficulty with language, and problems with familiar tasks. However, the subjective nature of these signs makes the task of naive judgments inefficient, highlighting the importance of pattern recognition in Electronic Health Record (EHR) clinical data over the period. In the past, the research community has tried to understand factors and biomarkers contributing to progression of dementia, but more work is needed to focus on variables’ contribution towards dementia onset [4, 30].

Several prior studies have employed learning-based mechanisms (machine learning and deep learning), transformers and open AI (Artificial Intelligence) models to detect and predict the progression of mild cognitive impairment (MCI) and dementia onset [11, 13, 21, 22]. The Time-aware Long Short-Term Memory (T-LSTM) neural network, a variation of LSTM (Long Short-Term Memory), is a well-known model that captures the time interval between two consecutive visits of a person to examine temporal changes in health status [2]. However, there is a lack of explanation regarding the decision-making process for early prediction of dementia using T-LSTM. Similarly, the other time-aware attention networks and their variants fail to deploy safe and responsible models for decision-making, suggesting the need to resolve the problem of uncertainty and explainability.

To address this problem, we performed network analyses to examine the changes in individuals’ health conditions in the prospective population-based cohort, Mayo Clinic Study of Aging (MCSA), from initial MCI diagnosis to dementia onset. We investigated how the associations among variables (i.e., health conditions) change from the incident stage of MCI to the development of dementia, explaining the dynamic nature of variable relationships. Furthermore, we examined the sex-specific impact of variables in relation to dementia, identifying differences between males and females.

Network analysis is a flexible method to explore associations among variables and text scores of EHR. After evaluating complexity in neuropsychological assessment, Tosi suggested that healthy subjects showed a segregated pattern, suggesting a good specificity of each test in measuring a specific cognitive function [35]. Another study used psychometric network analysis to model relationships between neurocognitive variables in cognitive normality (CN), amnestic mild cognitive impairment (aMCI), and early Alzheimer’s disease (eAD), indicating structural changes across different groups [10]. In this study, we choose to explore network analysis for finding correlations among different variables of HER data including chronic diseases, NPI-Q scores and intrinsic variables of disease.

## Methods

### Study design and subjects

This study was approved by the Mayo Clinic Institutional Review Board and the Olmsted Medical Center Institutional Review Boards. The study used data from the MCSA cohort [31]. The MCSA is a prospective population-based cohort study of cognitive aging with comprehensive periodic cognitive assessments repeated every 15 months. Eligible persons from the Olmsted County, Minnesota population, were randomly selected in an age- and sex-stratified manner and evaluated comprehensively in person by three independent evaluators (study coordinator, physician, psychometrist). A consensus committee comprised by three evaluators (i.e., study coordinator, physician, and neuropsychologist) reviewed the data for each participant and assigned diagnosis by consensus, using previously published criteria to diagnose the participant with MCI or dementia, or cognitively unimpaired. The MCSA cohort comprises 6,543 unique patients.

**Fig. 1:**
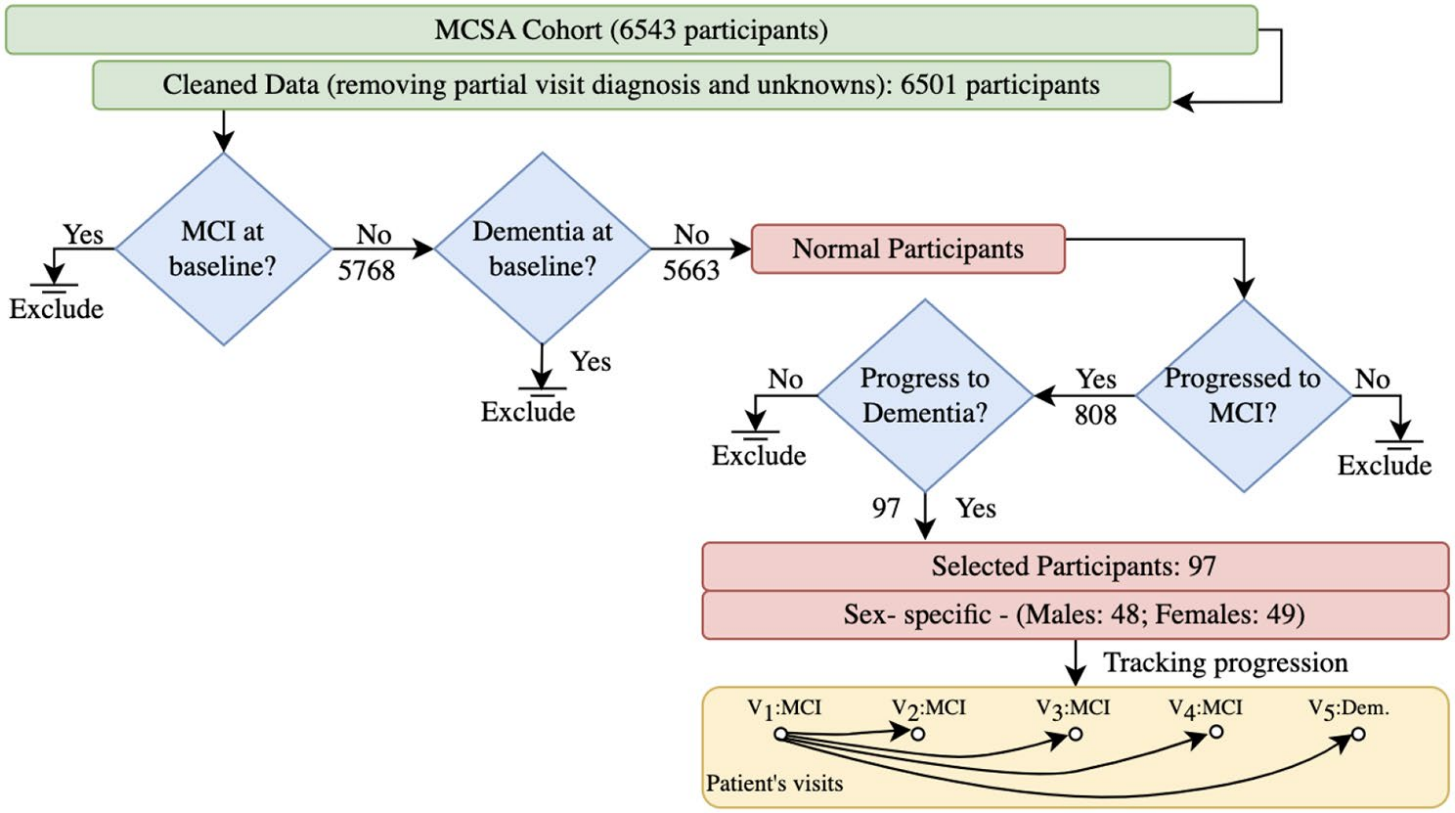
Sample selection for tracking progression from incident MCI to dementia onset

Figure 1 shows the process of study subject selection. We excluded participants who had MCI or dementia at the baseline (1st visit to MCSA) to not consider participants with existing MCI and dementia. Then, we selected the participants who developed MCI and progressed to dementia in later visits. We finally obtained 97 participants who visited MCSA more than one time since the first MCI diagnosis and progressed to dementia.

The inclusion criteria for this dataset stipulated that each participant had to attend at least one follow-up appointment after their diagnosis of MCI to facilitate the monitoring of disease progression over time.

### Data

In this section, we discuss nature of the data. The study population data, as presented in Table 1, showcases demographic information. The median age of the participants is 83.8-year-old with fairly even female and male. For the Apolipoprotein E e4 status, which is often associated with various health implications, less than half of the participants (42.2%) tested positive. Table 2 summarizes variables collected from the MCSA and used in network analyses. Furthermore, we remove variables that contain insufficient information for more than 50% of the instances for filtered data (see the final list of variables in the box below).

**Table 1:** Variables at baseline

Characteristics	N = 97
Age, median (IQR)	83.8 (79.2, 87.7)
Sex	
Female (%)	49 (50.5)
Male (%)	48 (49.5)
Apolipoprotein E e4 status	
Yes (%)	41 (42.2)
No (%)	56 (57.7)

**Table 2:** Variables at baseline

**Participants’ Demographics**
Age, Sex, Education (in years),
**Genotyping**
Apolipoprotein E e4 status (Yes, No)
**Participants’ Chronic Conditions**
Diabetes, Hypertension, Dyslipidemia, Atrial fibrillation, Angina chest pain, Myocardial infarction, coronary artery disease, Stroke, Peripheral Vascular Disease
**Cognitive and Functional sores**
Clinical Dementia Rating Scale global score (CDRGlob), Clinical Dementia Rating Sum of boxes (CDRSum), Functional Activities Questionnaire (FAQ)
**Neuropsychiatric symptoms**
Beck Depression Inventory-II, Beck Anxiety Inventory, NPI-Q: Delusions, Hallucinations, Agitation, Depression/Dysphoria, Anxiety, Euphoria/elation, Apathy/Indifference, Disinhibition, Irritability/lability, Motor Behavior, Appetite/eating change

We included all the visits of a participant, after the first MCI diagnosis to the first dementia diagnosis. Different participants have different number of visits because some were recruited later, and some missed certain visits (ranging from 2 to 7 visits per participant). The timing of MCI to dementia progression varies due to different trajectories of relevant health condition (ranging from 2 to 7 visits from MCI to dementia).

### Fundamentals of graph theory

A graph is a mathematical representation of a set of objects where some pairs of the objects are connected by links. These objects are often referred to as nodes or vertices, and the links between them are called edges or arcs. Graphs are used to model relationships between various entities, such as medical conditions. We investigated the progression of MCI to dementia for patients at different time intervals through graph learning.

We construct an edge between two nodes if their Pearson Correlation Coefficient (PCC) is above a certain threshold. The PCC measures the strength and direction of the relationship between the two variables, where a positive value indicates a positive correlation, and a negative value indicates a negative correlation. The PCC is calculated as r to compute the pairwise correlation among variables as shown in Eq. 1:

1 $$\:r\:=\:\:\frac{(N\sum\:{V}_{i}{V}_{j}\:-\:(\sum\:{V}_{i}\left)\right(\sum\:{V}_{j}\left)\right)}{\sqrt{(N\sum\:{V}_{i}^{2}\:-\:{\left(\sum\:{V}_{i}\right)}^{2})\:(N\sum\:{V}_{j}^{2}\:-\:{\left(\sum\:{V}_{j}\right)}^{2})}}$$

where $\:N$ is the total number of nodes ($\:\left|V\right|$) in the network. Here, $\:{V}_{i}$ and $\:{V}_{j}$ represents the node degrees. The *node degree* in a graph refers to the number of edges connected to that node [12]. We used Pearson correlation to find positive and negative linear relationships between variables in each phase, as supported by existing studies [7, 9, 27].

#### Assortativity

Assortativity is a measure of the tendency of nodes in a graph to connect to other nodes that are like themselves in some way and is denoted by A in this manuscript. It measures the correlation between the degrees of nodes at either end of an edge in the graph. It ranges from − 1 to 1, with a positive number indicating a tendency for similar nodes to connect, and a negative number indicating a tendency for dissimilar nodes to connect. Thus, positive assortativity indicates that nodes with high degrees tend to be connected to other nodes with high degrees, while negative assortativity indicates that high-degree nodes tend to be connected to low-degree nodes. Assortativity can be thought of as a measure of homophily or the tendency of like to connect with like.

#### Community detection

Community detection is the process of identifying densely connected groups or sub-networks of nodes within a larger network/graph. The goal of community detection is to partition the nodes in the graph into groups (i.e., communities) that are more densely connected internally than with the rest of the graph. For Graph G, community detection is used to identify groups of variables that are more closely related to each other than to other variables in the graph.

### Graph construction

Our study has two states, MCI, and dementia. Consider a set of patients $\:{P}_{i}=\{{P}_{1},\:{P}_{2},{P}_{3},\dots\:,\:{P}_{n}\}$ where $\:i$ is the number of patients in $\:P$. Each patient $\:{P}_{i}$ is represented as a unique ‘CLINIC ID’ in the MCSA dataset, that visits over the period of$\:\:T=\{{T}_{1},\:{T}_{2},\dots\:,\:{T}_{m}\}$ where *m* is the total number of visits for every patient $\:{P}_{i}$.

Consider a graph $\:G\:=\:(V,\:E)$, where $\:V$ is the set of nodes representing the variables in the MCSA data and E is the set of edges representing the relationships between these variables. In this work, $\:\left|V\right|\:=\:29$ and $\:\left|E\right|$ depends on the extent of connection between two variables.

### Network analysis using graph theory

**Fig. 2:**
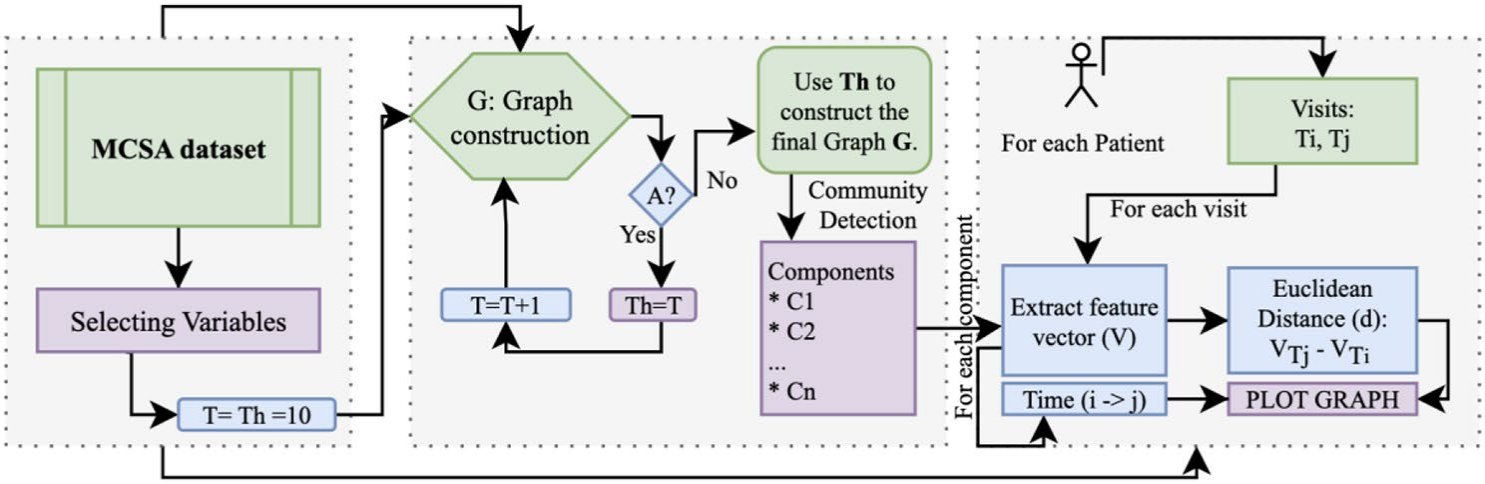
Overview of the proposed approach. Here T represents the threshold we increment by 1 to check the graph’s Assortative (A) nature

We aim to investigate the progression of dementia onset for patients with varying number of visits at different time intervals through graph learning. However, as an initial step of this study, we choose to represent the progression of dementia by identifying the most correlated variables. The overview of our proposed framework is given in Fig. 2 where we select a group of patients’ visits from the MCSA dataset to examine the patterns in progression from the first MCI visit to dementia. We identify threshold (Th) by iteratively increasing the value with the condition of acquiring the threshold value that supports maximal correlation with assortative nature of the graph. Details are provided in the next section. Next, the progression of dementia is witnessed with successive visits.

The overview of our methodology is given as follows:

1. We first applied the Pearson Correlation Coefficient (PCC) to construct the graph-representation where nodes are the variables and correlation is illustrated by edges between these variables.
2. We identified the threshold value for community detection through the network models for three forms of dataset: (i) the visit of the first MCI diagnosis (MCI-V), (ii) the visit(s) between MCI and dementia (M2D-V), and (iii) the visit of dementia diagnosis (DEM-V).
3. We presented the progression of dementia from the first MCI diagnosis at MCSA and its progression over the period.

### Finding associations

The correlation coefficient defines the extent of close associations among health conditions that participants have. For instance, how closely the Hypertension is associated with Diabetes. For all three groups, we associate these attributes using Pearson Correlation Coefficient. We chose this approach because PCC measures the linear relationship between different health conditions. Unlike cosine similarity, which measures the cosine of the angle between two vectors in a multi-dimensional space and is often used to understand the orientation or similarity in orientation between two, PCC highlights the strength of a linear relationship. When constructing a network of associated health conditions, using PCC helps to filter out connections that might occur by chance, particularly when setting a threshold for significance. Thus, we examine optimum threshold value for further network analysis.

We then find the context-aware threshold to find significant correlations and construct a graph among attributes by establishing connections (among attributes). The value of threshold is obtained by iterating it until just before the graph is disassortative [28]. We use the grid-search method to find threshold value by retaining the most correlated elements through iterative decomposition and assortative check of the resulting graph; the value of r ranges from − 1 to 1, where − 1 represents a perfect negative correlation, 0 represents no correlation, and 1 represents a perfect positive correlation. The higher value of threshold signifies strong relations among variables. The graph representation of a research question of progression to dementia onset is highly efficient for assortative networks having a positive assortativity coefficient. Assortativity captures the tendency of similar nodes (attributes) to connect for understanding the meaningful relationships and clusters of health-related attributes. LASSO penalization may eliminate weak but meaningful connections, potentially losing important information in the limited network of attributes. Thresholding Based on Correlation or Mutual Information is sensitive to noise and may retain spurious connections or remove important ones based on the chosen threshold.

To identify relationships and dependencies between variables, we employ assortativity which allows us to limit the iterative increase in threshold values and preserve the underlying semantics of the correlated variable driven network [34]. To optimize the threshold value, we keep increasing the threshold value and a check on the assortative nature of the graph. As we increase the threshold value, we retain the stronger connections in the resulting graph, monitoring the nature of the graph to identify optimal threshold value. This step enables us to identify the optimal threshold values that lead to the most meaningful and informative attribute network.

### Detecting communities

Identifying communities in Graph G is important for tracking the progression of dementia onset because it can help to identify sets of variables that are strongly correlated and may be involved in common biological processes or pathways. Thus, community detection can assist in creating new ways to diagnose and treat dementia and enhance our comprehension of the biological processes involved in the disease. By identifying the communities of variables, the clinical researchers can better understand the complex interplay among different variables and how they contribute to dementia risk and progression.

In this study, our goal was to uncover the structure of communities within the network of health conditions. Specifically, we sought non-overlapping communities to clearly delineate the distinct groups of health conditions that cluster together. This distinction is vital as it provides insights into conditions that tend to co-occur, aiding in a better understanding of disease comorbidities.

Given this context, we opted for undirected graphs because the mutual influence between two health conditions does not inherently possess a directionality; the occurrence of one does not unilaterally affect the other, but rather they tend to co-exist in a patient’s profile. We compute three networks: (i) one for all the first visit of MCI diagnosis, (ii) one for all the visits between the first visit of MCI diagnosis and the first visit of dementia diagnosis, (iii) one for all the first visit of dementia diagnosis.

We chose to implement unweighted models once we construct graph for each of these three networks using the threshold for PCC. Our preliminary analysis suggested that the strength of association, or the weight between any two conditions, did not significantly alter the community structure for the scope of our study. This simplification allowed for clearer community boundaries and more straightforward interpretability of the resulting network structures. Thus, we use two well-established community detection models: (i) Girvan Newmann Algorithm [16], and (ii) Clauset–Newman–Moore (CNM) [5], to identify groups of variables that tend to co-occur frequently. A key difference between Girvan Newmann Algorithm uses the predefined number of clusters but the CNM algorithm does not.

The Girvan-Newman algorithm detects communities in complex networks progressively removing edges from the network in order of their “betweenness” centrality until the network breaks down into smaller communities [16]. The betweenness centrality of an edge is a measure of the number of shortest paths between pairs of nodes in the network passing through that edge. Betweenness centrality (B) is widely used to measure how much a node or edge influences communication or interaction flow in the network as shown in Eq. 2.

2 $$\:B\:\left({V}_{i},\:{V}_{j}\right)=\:{\sum\:}_{s\:\ne\:\:t}\frac{{\sigma\:}_{\left\{s,t\right\}}\:\left({V}_{i},\:{V}_{j}\right)}{{\sigma\:}_{\left\{s,t\right\}}}$$

where $\:({V}_{i},{V}_{j})$ is an edge from $\:{V}_{i}$ to $\:{V}_{j}$, $\:{\sigma\:}_{\left\{s,t\right\}}$ denote the source and target in the network, $\:{\sigma\:}_{\left\{s,t\right\}}\:({V}_{i},\:{V}_{j})$ represents the number of links passing through an edge $\:({V}_{i},\:{V}_{j})$ while traversing from source $\:s$ to target $\:t$ against the ones which do not pass through $\:({V}_{i},\:{V}_{j})$. The edges with high betweenness centrality are more likely to connect different communities, and thus removing them is more likely to break up the network into distinct communities.

**Fig. 3:**
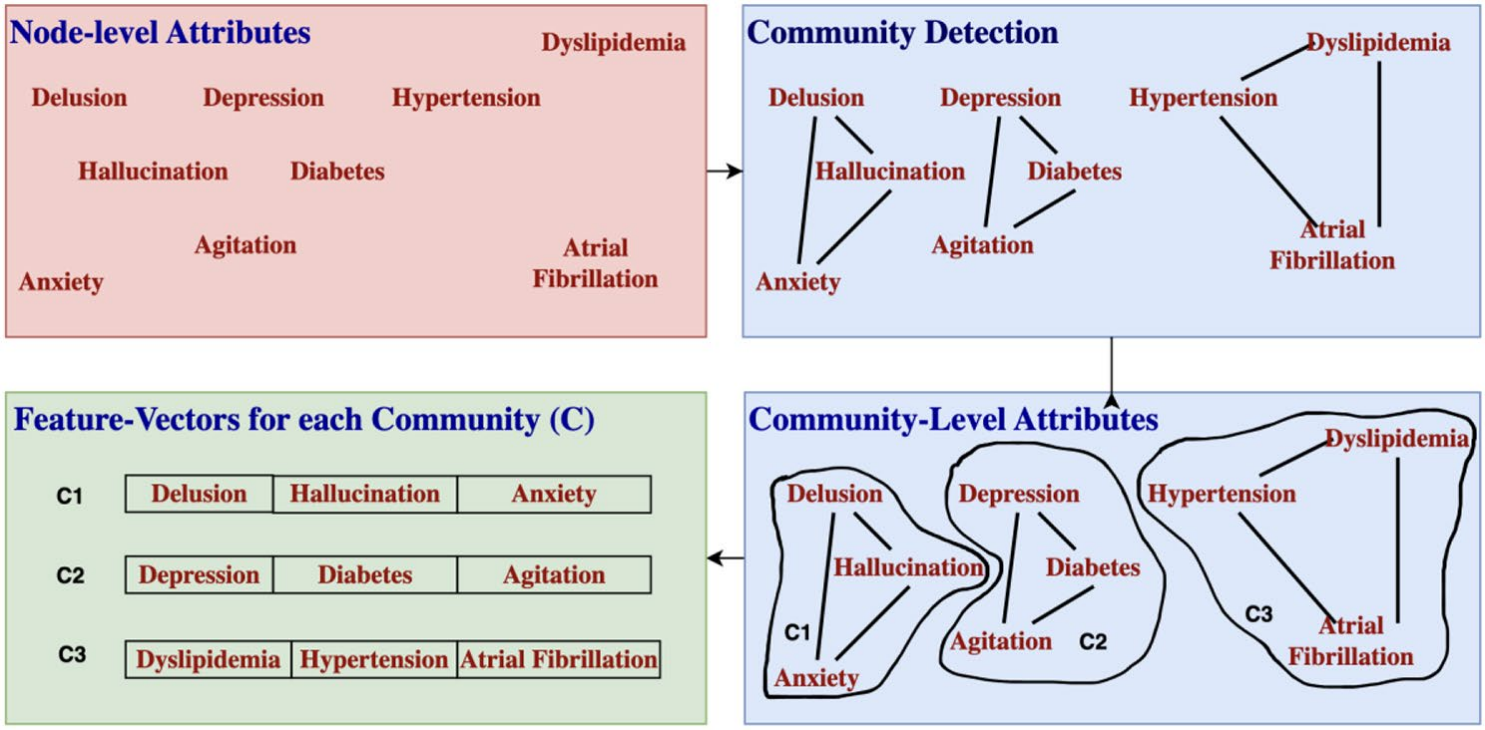
An illustration of transition from node-level attributes to community-level attributes, followed by feature vectors

The CNM algorithm is a popular method for community detection in networks, based on greedy modularity maximization [5]. The algorithm works by iteratively merging nodes into communities that increase the modularity of the network. It begins by assigning each node to its own community and then iterates over all pairs of nodes and calculates the change in modularity that would result from merging them into a single community. The algorithm greedily merges the pair that results in the largest increase in modularity and repeats this process until no further improvement in modularity can be achieved. We finally obtain community-level attributes as shown in Fig. 3.

### Characterizing progression

To simplify the analyses, we focused on characterizing the progression of dementia examining variables in: (i) the visit of the first MCI diagnosis (MCI-V), (ii) the visits between MCI and dementia (M2D-V), and (iii) the visit of dementia diagnosis (DEM-V) through batchwise analysis of community detection. We examined the progression of variables in different communities for MCI-V, M2D-V, and DEM-V by identifying feature vectors and distance between them in cohort visits.

A feature vector is defined by a set of variables to track the progression of change in the characteristics of the patients’ visit. Consider $\:C=\{{C}_{1},\:{C}_{2},\dots\:,\:{C}_{k}\}$ as the set of $\:k$ communities obtained after deploying the community detection algorithms. Every community $\:{C}_{v}$ consist of different values for V$\:=\left\{{v}_{1},\:{v}_{2},\dots\:,\:{v}_{l}\right\}$, where $\:l$ may vary for every community $\:{C}_{v}$. V represents the feature vector corresponding to each community of health conditions. We considered a set of variables in a community as a feature vector.

We first normalize our data using min-max normalization policy. We calculate the distance between feature vectors whose values are the values of attributes in each community obtained from two separate visits. We employ distance measurements to assess the importance of variations among participants’ visits, based on a collection of variables. Our hypothesis suggests that analyzing these patterns of differences could offer valuable insights into whether individuals are maintaining mild cognitive impairment (MCI) or transitioning toward dementia. This enables us to compare changes in the variables over time and assess the patient’s overall progression. By utilizing this approach, we aim to gain insights into the dynamics of the variables within each community and to identify potential trends or patterns that may be indicative of dementia onset progression. Here, we define distance $\:Dist{C}_{a}$ as Eq. 3:

3 $$\:Dist{C}_{v}\:={V}_{{T}_{j}}\:-\:{V}_{{T}_{i}}$$

where $\:{V}_{{T}_{j}}$ is the feature vector for $\:{j}^{th}$ visit and $\:{V}_{{T}_{i}}$ is a feature vector for $\:{i}^{th}$ visit of a patient. Here $\:{T}_{j}$ belongs to [M2D-V, DEM-V]; and $\:{T}_{i}$ belongs to [MCI-V]. To measure distance, we use Euclidean distance.

## Results

### Network associativity

Table 3 contains the characteristics of our network analysis study with three separate groups of datasets, labeled as MCI-V, M2D-V, and DEM-V, representing separate phases associated with cognitive impairment or dementia.

**Table 3:** Threshold values, assortative coefficient, and clustering coefficient in the network

	MCI-V	M2D-V	DEM-V
Threshold	25%	30%	21%
Assortativity Coefficient	0.053	0.093	0.216
Clustering Coefficient	0.614	0.577	0.640

Threshold represents a cutoff point used to define or distinguish connections in the network being analyzed. For every phase, we initiate the threshold value as 10% to construct the graph G and increase the value by 1% until network becomes non-assortative. Assortativity Coefficient is a measure of how many nodes (i.e., variables) in a network tend to connect with other nodes that are like them. The assortativity coefficient increases from MCI-V to DEM-V, suggesting that variables are more likely to connect (or share similar characteristics) with other variables as the cognitive decline progresses.

The threshold value, 30% for MCI to Dementia visits signify strong relationships among characteristics of variables in the network for the M2D-V stage. Yet, post the dementia diagnosis visit, the intricacy of these relationships intensifies and complicated, with a minimal threshold value of 21%, indicating an early halt due to the graph’s shift towards non-assortativity.

Clustering Coefficient measures the degree to which nodes (variables) in the network cluster together or form interconnected groups. A high clustering coefficient indicates that a node’s neighbors are also neighbors of each other. The clustering coefficient is highest in DEM-V and lowest in M2D-V indicating that the denser clusters among variables in the DEM-V group as compared to the other phases. In DEM-V, the variables interrelate or co-occur closely with each other, implying the association among them in terms of the cognitive decline process. The network may contain certain tightly knit groups or communities of variables that are intricately related in the context of cognitive decline. These communities represent specific aspects or dimensions of the cognitive decline process. With the high clustering coefficient for DEM-V, the interactions among variables become more complex or unpredictable because of more connections and uncertainty in the patterns. This finding aligns with the previous research that may complicate early prediction efforts in dementia using computational intelligence [8]. The lowest average clustering coefficient in M2D-V suggests either the variables operate more independently or the network is modular. However, this variation might be due to multiple visits (2 to 7 visits) for each patient in M2D-V, compared to a single visit in MCI-V and DEM-V. As such, we notice the redundancy in the values of variables due to similar state of a person (MCI) in multiple visits, suggesting variation in correlation coefficient.

**Fig. 4:**
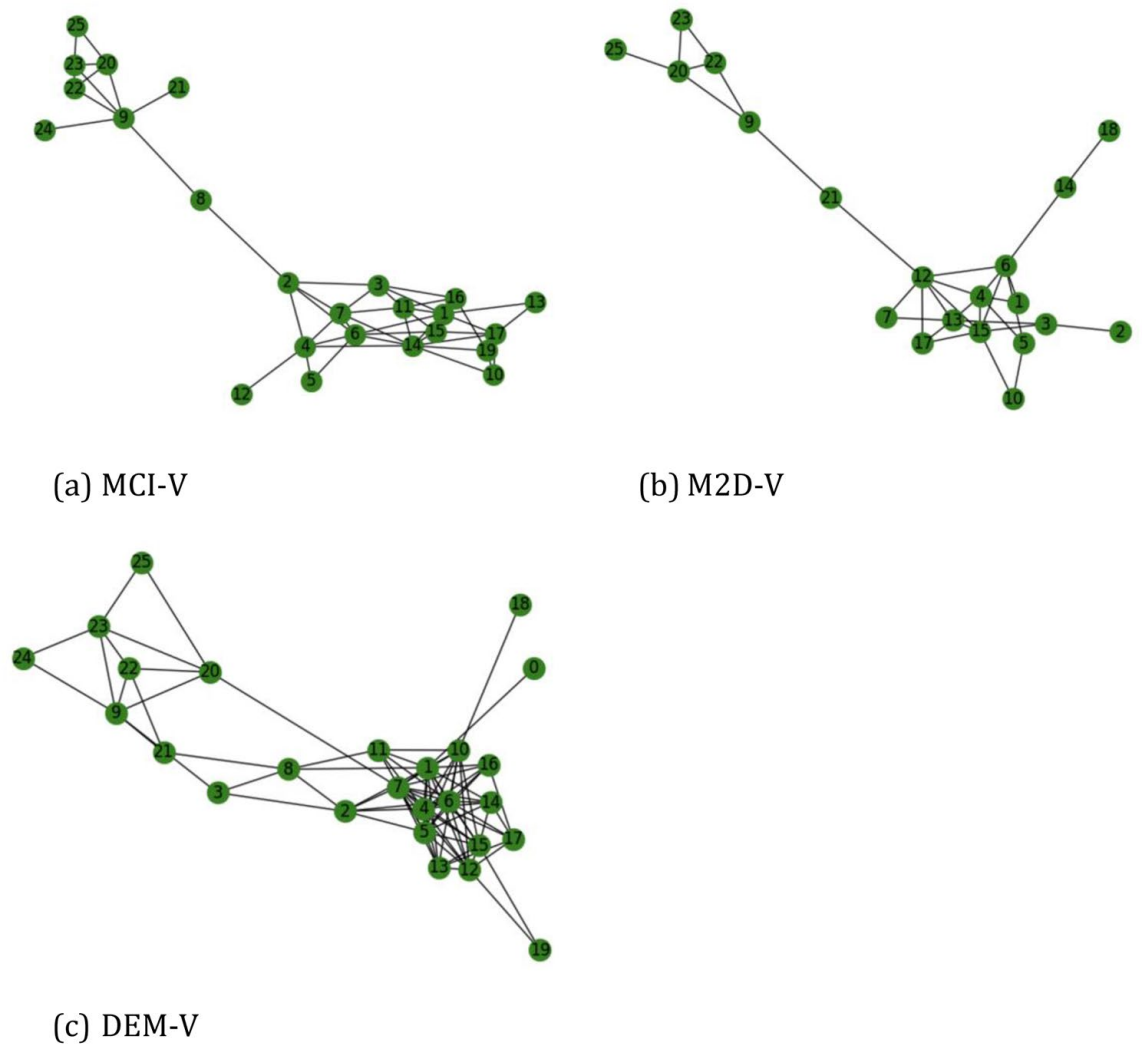
Network Associativity for (**a**) MCI-V (top-left) (**b**) M2D-V (top-right) and (**c**) DEM-V (bottom-left). (0: Apolipoprotein E e4 status, 1: Motor Behavior, 2: Beck Depression Inventory-II, 3: Beck Anxiety Inventory, 4: CDRSum, 5: CDRGlob, 6: Functional Activities Questionnaire, 7: Depression/Dysphoria, 8: Diabetes, 9: Dyslipidemia, 10: Delusion, 11: Hallucination, 12: Agitation, 13: Anxiety, 14: Apathy/ Indifference, 15: Disinhibition, 16: Appetite/eating change, 17: Irritability/lability, 18: Peripheral Vascular Disease, 19: Euphoria/elation, 20: Angina chest pain, 21: Hypertension, 22: Coronary artery disease, 23: Myocardial infarction, 24: Stroke, 25: Atrial fibrillation)

Figure 4 shows the associations among different variables for MCI-V, M2D-V, and DEM-V. We observe the changing association of certain health conditions during MCI-V, M2D-V, and DEM-V that aligns with the past study [37] reporting health conditions such as diabetes, hypertension, atrial fibrillation, cardiovascular, cataracts, depression, anxiety, and mobility impairments associated with cognitive decline. In MCI-V, depression is associated with anxiety, hallucination, Clinical Dementia Rating Scale global score (CDRGlob) and Functional Activities Questionnaire score, indicating correlation among these variables. However, in M2D-V, depression is directly associated with agitation and anxiety and indirectly associated with CDRSum and disinhibition with one hop. In DEM-V, depression is associated directly with all the above and CDRGlob. The CDRGlob shall vary from MCI-V to DEM-V and will associate with the other variables to a different extent, depicting significant variation during DEM-V and its correlation with highly participating variables towards dementia. Additionally, the isolated nodes (variables) having the least connection with the other variables, such as diabetes in M2D-V, have no variation and remains same. However, diabetes has a low clustering coefficient in MCI-V and high clustering coefficient in DEM-V indicating more correlation with other variables. Similarly, hypertension is associated with dyslipidemia in MCI-V, followed by an additional association with agitation during M2D-V. Hypertension is associated with anxiety, dyslipidemia, diabetes and coronary artery disease in DEM-V and all these conditions are associated with dementia as reported in the past studies [37].

### Network community

**Fig. 5:**
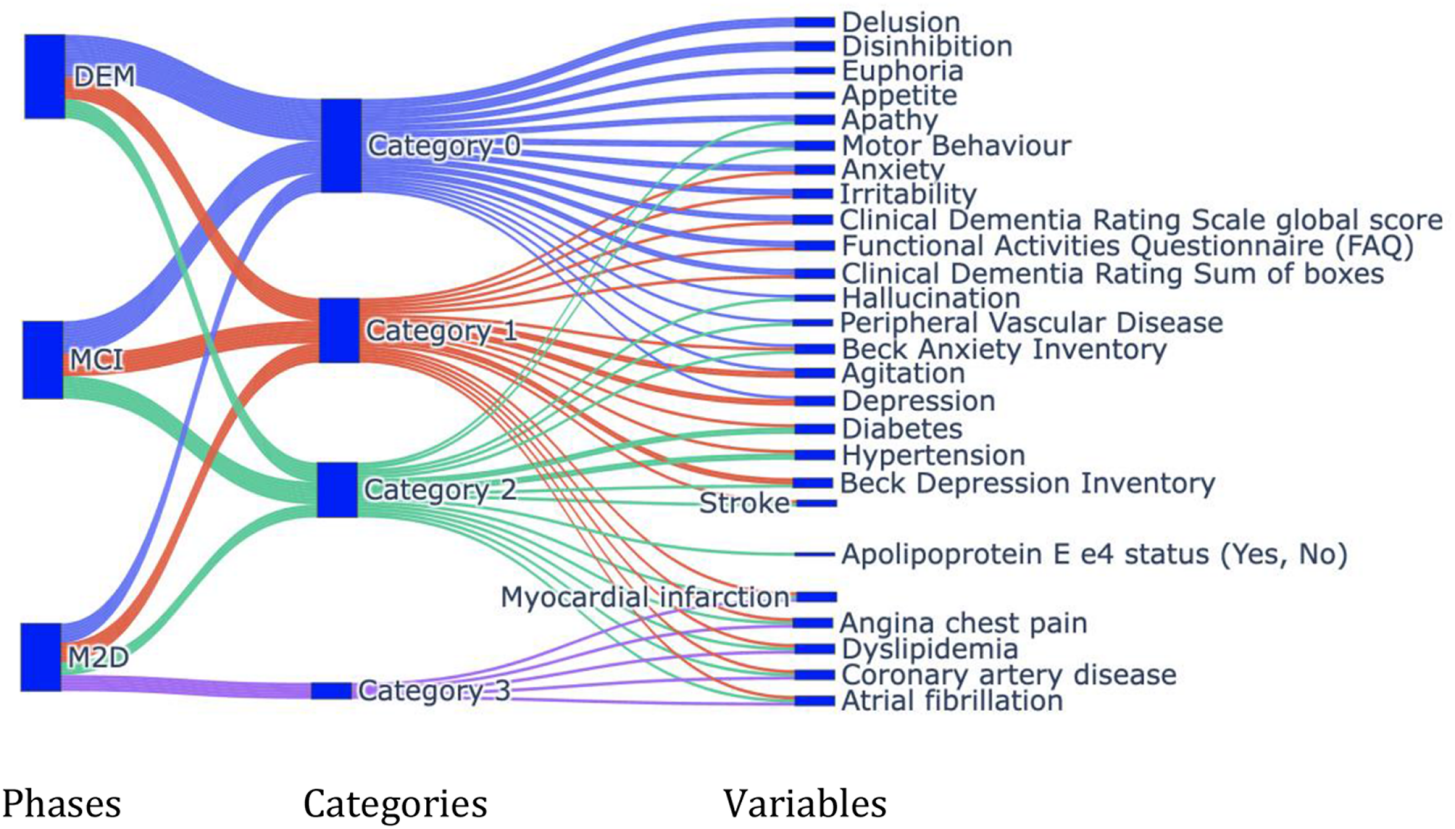
Association among variables changes in network communities for three distinct phases: MCI-V (MCI), M2D-V (M2D), and DEM-V (DEM). Different colors represents the distinct community and the corresponding variables in that category. Here, category 0 to 3 are the communities of variables, evolved for different groups. We observed the clusters of different variables suggesting associations with MCI onset”

A particular set of variables may be associated with each other at the early stage of cognitive impairment, but their associations may change as the cognitive decline progresses. The evolving nature of the associations among variables highlights the need for a dynamic approach that tracks the changes over time to effectively characterize the progression to dementia onset. Network communities of variables illustrates changes in associations among these variables for MCI-V, M2D-V, and DEM-V groups. We obtained 3, 4, and 3 communities using Girvan Newmann community detection algorithm for those three groups and represent the associations among variables in Fig. 5.

We find common variables across communities in all three groups during progression of cognitive decline and categorize them into four different categories. Categories are determined based on the dominating variables. Many variables do not change significantly in M2D-V as it contains multiple visits with prevailing health conditions thus producing more communities than the other groups. Category 0 (blue lines) has many variables associated with each other. Similarly, orange, green, and purple lines indicate the common variables merging into categories 1, 2 and 3, respectively.

We observe neuropsychiatric symptoms (NPI-Q) in category 0 and CDR, cognitive and Functional scores appear in both category 0 and 1. Category 1 also includes agitation and depression. Chronic diseases, such as diabetes and hypertension, belong to category 2, depicting corresponding higher correlations as compared to the other variables. Such correlations suggest the association among variables at various stages of cognitive decline, showing the complex nature of dementia. Some variables (angina, chest pain, coronary artery disease, atrial fibrillation, dyslipidemia, and myocardial infarction) occur together in multiple categories, suggesting high correlation.

While high association among variables in a dataset may suggest a relationship between them, it does not necessarily imply that they contribute significantly to the progression to dementia. Further examination is necessary to determine the impact of each variable in the progression to dementia to gain a more nuanced understanding of its underlying mechanisms.

### Characterizing community variables from MCI to dementia

**Fig. 6:**
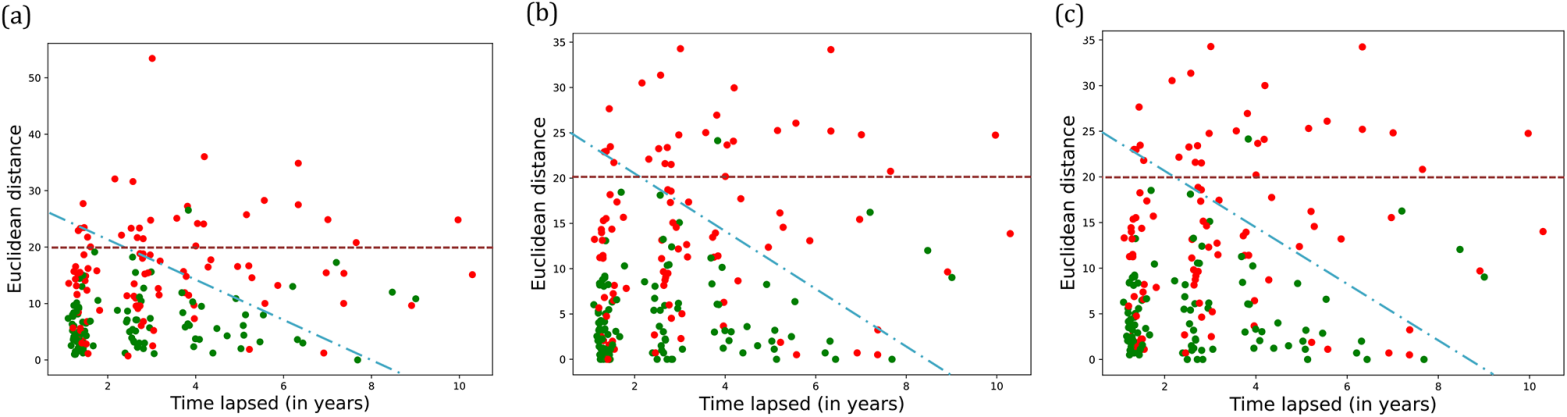
Changes of community variables from MCI to dementia onset. Green dot denotes that the participant is still MCI and red dot denotes that participant has progressed to dementia. Euclidean distance above the maroon dotted line reflects a high chance of dementia (> 90%). The visits below the blue dashed line are mostly mixture of MCI and dementia with a relatively short distance. (**a**) Community in MCI-V (corresponding variables: Beck Depression Inventory, CDRGlob, CDRSum, Diabetes, Agitation, Depression, Functional Activities Questionnaire (FAQ)). (**b**) Community in M2D-V (corresponding variables: CDRGlob, CDRSum, Delusion, Disinhibition, Motor behavior, Functional Activities Questionnaire (FAQ)). (**c**) Community in DEM-V (corresponding variables: CDRGlob, CDRSum, Agitation, Anxiety, Apathy, Appetite, Delusion, Depression, Euphoria, Irritability, Peripheral Vascular Disease, Functional Activities Questionnaire (FAQ))

We examined changes in variables of network communities over time as the patients progressed to dementia to provide useful insights on the impact of variables to dementia progression. We selected the most representative community in each stage and plotted the distance-time graphs as the patient progresses towards dementia (Fig. 6). The most representative community is a cluster of variables representing significant changes among variables for successive visits, highlight patterns for those progressing towards dementia or stay in MCI. We use Euclidean distance metric to monitor changes in the variables and identify the most contributing variables towards dementia onset through statistical analysis. The higher the value of the Euclidean distance, the more the change of variables towards progressing to dementia over the period. The x-axis denotes the time lapse between the visits ranging from the first visit of MCI diagnosis to the visit of dementia onset, and the y-axis denotes the Euclidean distance between feature vectors (variables in communities) reflecting a change of variables in a community for a given time lapse. The blue dotted line Fig. 6, represents the intercept formed by the linear equation, bounded by x-axis and y-axis covering the most cases where a person stays in MCI for given time-lapse. It is the bounded area with linear equations: 25x + 9y = 225, y > = 25, x > 9. We identified the intercepts: (i) the line that intersects x-axis at (a, 0) and (ii) the line that intersects y-axis at (0, b). Here b is the values of Euclidean distance and a is the time lapse. The equation becomes:

4 $$\:\frac{\text{x}}{\text{a}}+\frac{\text{y}}{\text{b}}=1$$

Which can be represented as $\:\text{b}\text{x}+\text{a}\text{y}=\text{a}\text{b}.$ We vary values for a and b such that the resulting linear equation optimally covers maximum green dots (participants visits where they are still MCI).

There is more mixture of red and green dots (dementia and non-dementia) in the beginning of time laps (close to ‘0’ in the x-axis) compared as the time progresses. While we are unable to determine the precise time-lapse for dementia onset, time-lapse before dementia ranges from approximately 1 year to beyond 10 years. We found that distance > 20 showed high chance of progression to dementia during early stages post MCI diagnosis (i.e., 90% of people who have distance > 20 progressed to dementia).

The Euclidean distance correlates with the progression to dementia; the distance greater than 20 (dotted line in Fig. 6) reflects a high probability (> 90%) of dementia, between 10 and 20 reflects a moderate chance of dementia (70%). On observing the common variables among these three sets, we obtain variables assessing cognitive and functional impairment (CDR and FAQ) to be the most significant variables in progression to dementia. These observations support existing studies on early prediction of dementia and Alzheimer’s disease [36]. Staying in MCI without progression to dementia has a lower value of Euclidean distance (i.e., not much changed variables) associated with disinhibition, delusion, and motor behavior. It suggests that these variables may relate to dementia progression from MCI. Our study findings indicate the importance of recognizing minor alterations in daily activities in future longitudinal studies to investigate different pathways from normal cognitive aging to the cognitive decline characterizing various stages of Dementia.

Furthermore, we selected the variables from our observations in Fig. 6 by considering its presence in majority (two out of three) cases, namely, delusion, Clinical Dementia Rating, functional activities questionnaire score, and agitation. We make the following observations:

**Table:** 

	Stats	CDRGlob	CDRSum	FAQ	Delusion	Agitation
All visits	*Mean*	*0.61*	*2.87*	*8.00*	*0.07*	*0.11*
	*S.D.*	*0.52*	*3.18*	*8.53*	*0.26*	*0.313*
MCI-V	Mean	0.36	1.02	3.17	0.03	0.05
	S.D.	0.22	0.99	4.62	0.17	0.22
M2D-V	Mean	0.46	1.89	5.60	0.05	0.12
	S.D.	0.23	1.55	5.83	0.23	0.32
DEM-V	Mean	1.03	5.78	15.44	0.13	0.15
	S.D.	0.70	3.84	9.08	0.34	0.36

In the above Table, we observe that the mean values and standard deviation of all five variables: delusion, Clinical Dementia Rating, functional activities questionnaire score, and agitation, increases as participants move from MCI to Dementia, suggesting change in variables during MCI onset.

### Sex-specific community variable changes

**Fig. 7:**
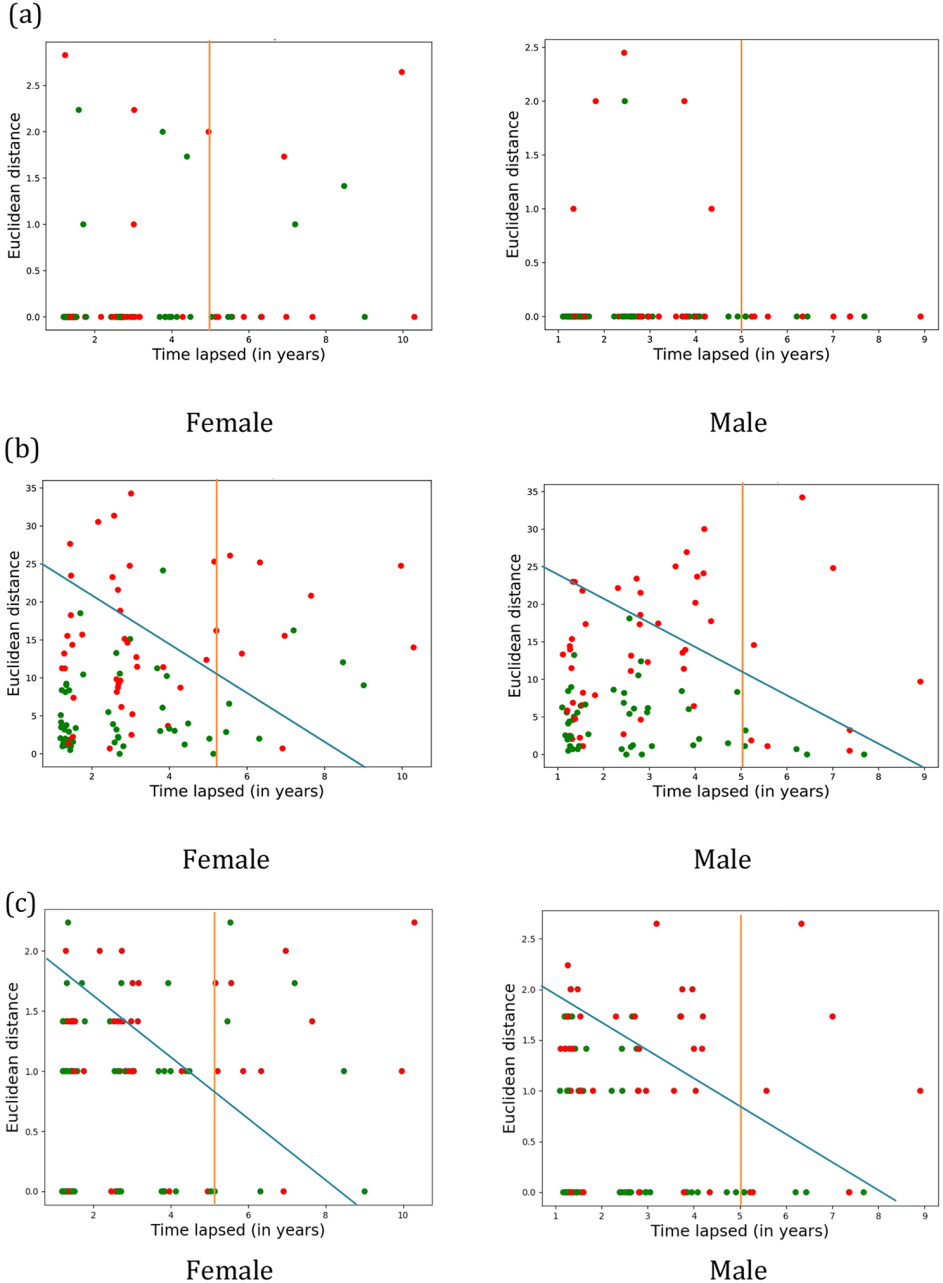
Sex-specific changes of community variables with time-lapse. Green dot denotes that the participant is still MCI and red dot denotes that participant has progressed to dementia. (**a**) Community that contains variables: Atrial fibrillation, Angina chest pain, Coronary artery disease, Dyslipidemia, Hypertension, Myocardial infarction, Stroke. (**b**) Community contains cognitive and functional impairment (CDR and FAQ) variables and NPI-Q scores: CDRGlob, CDRSum, Agitation, Anxiety, Apathy, Appetite, Delusion, Depression, Disinhibition, Euphoria, Irritability, Peripheral Vascular Disease, Functional Activities Questionnaire (FAQ). (**c**) Community that contains variables for neuro-psychiatric characteristics: (Anxiety, Apathy, Appetite, Delusion, Disinhibition, Euphoria, Hallucination, Irritability, Motor behavior)

We examined changes in variables of network communities over time between female and male participants in the MCSA. Figure 7 shows the more changes among females as compared to male participants with increased time-lapse. Furthermore, the blue division suggests the dementia is more certain with increased time-lapse and low Euclidean distance among variables of the community for male participants. In general, we observe that the number of visits by female participants is more than those of male participants after 5 years of MCI-V. We observed different patterns for following three communities:

1. *Chronic conditions*: We consider the community of variables (Atrial fibrillation, Angina chest pain, Coronary artery disease, Dyslipidemia, Hypertension, Myocardial infarction, Stroke) in Fig. 7(a). Our research findings indicate that 26% of the visits in female participants (50% suggesting MCI and 50% suggesting dementia), after 5 years of first MCI diagnosis, have substantial Euclidean distance. However, among male participants, there is zero Euclidean distance between consecutive visits after 5 years of the MCI-V, suggesting increased uncertainty in predicting cognitive status among female participants due to change in chronic conditions.
2. *Cognitive and functional impairment (CDR and FAQ) variables and NPI-Q scores*: Next, we consider CDR and FAQ variables (CDRGlob, CDRSum, Agitation, Anxiety, Apathy, Appetite, Delusion, Depression, Disinhibition, Euphoria, Irritability, Peripheral Vascular Disease, Functional Activities Questionnaire (FAQ)). Our study reveals that in Fig. 7(b) where blue line holds threshold for trade-off between time-lapsed and Euclidean distance. We obtain the unbounded area with linear equations: $\:25x+9y=225,\:y>=25,\:x>9$, as a two-dimensional space suggesting the prospective decision-making for cognitive status. Although there is no uncertainty among male participants, however, 17% of the visits within the recommended space among female participants suggests MCI in-place of dementia, as cognitive status. Furthermore, if we consider participant visits after 5 years of MCI-V, we observe the visits two-fold, showing Euclidean distance (D) (i) D <= 5, (ii) D > 5. We observe only 21% of visits having D < = 5 among female participants, suggesting more visits with significant changes among CDR and FAQ variables even after 5 years as compared to 70% visits of male participants accounting for D < = 5. For D > 5, the cognitive status shows 1:3 ratio for cognitive status (MCI: dementia), showing no direct observations in the decision-making process for female participants. However, if the visits show high Euclidean distance, it indicates dementia as a cognitive state with high probability.
3. *Neuropsychiatric characteristics*: Furthermore, we examine the community that contains variables for NPI-Q: (Anxiety, Apathy, Appetite, Delusion, Disinhibition, Euphoria, Hallucination, Irritability, Motor behavior) in Fig. 7(c). Our research highlights distinct patterns in female participants with 64% of the visits after 5 years of MCI-V showing substantial change in the variables, as compared to the male participants with only 40% of the visits showing change in the variables during consecutive visits. Higher values of Euclidean distance for male participants gives more confidence in dementia state as compared to the female participants.

## Discussion

The analysis of individuals’ health statuses, using a network model to trace from the incident MCI to the onset of dementia, yielded notable observations. Diverging from conventional methods, our approach not only provides visual insights but also elucidates the underlying rationale. Through this study, we can (1) unravel the dynamic interplay among variables during the three distinct phases: MCI-V, M2D-V, and DEM-V, (2) identify key variables that influence the progression to dementia, and (3) explore the distinct impact of variables on dementia onset specific to each sex.

By uncovering the complex interplay among variables, we may identify potential targets for intervention and devise personalized treatment strategies that consider the individual patient’s unique circumstances. We explained notable findings regarding variables through Figs. 6 and 7. These figures are intended to show “changes of community variables” in different stages (MCI, MCI to Dementia, and Dementia Visit) as a whole. We reported notable finding of variables in the communities through qualitative analysis compared with existing literature (Fig. 6) and sex-specific community variable changes (Fig. 7) analyzing focused variables such as chronic conditions and neuropsychiatric characteristics.

By investigating the communities within the network and tracking their changes over time, we can detect early indications of dementia progression. For example, consider a community where certain variables prevail across all phases of dementia progression, such as CDRSum, CDRGlob, Functional Activities Questionnaire (FAQ). In contrast, other variables change as the progression moves from MCI-V to DEM-V.

In the context of dementia progression from MCI, prior research has highlighted the potential of treatments targeting neurological symptoms, like depression, to slow the advancement of the disease [6]. Our results align with these insights, further emphasizing the significant role neurological factors have in the progression of dementia. Addressing these risk factors over the period can indeed serve as an effective strategy to impede the disease’s trajectory.

Earlier research has shown a greater tendency for females to transition from MCI to dementia, a conclusion consistent with our own observations [32]. This variation in findings across earlier studies has led to the identification of distinct risk factors for each sex [3]. Our study showed these sex-specific risk factors using a community-based approach among variables over the time. Visual representations of these dynamic changes offer a better understanding of the various phenotypes exhibited by individuals with cognitive impairment. For example, based on the age-related time-lapse, patterns with green dot and red dots in Figs. 6 and 7 represent the state of cognitive impairment (MCI or DEM) within each community of variables.

Several studies examined the interaction between sex and various variables to understand how they collectively contribute to the development, progression, and manifestation of dementia [33]. Our study revealed notable changes in variables of three network communities with chronic conditions, NPI-Q, and NPI-Q with neuropsychiatric variables over time between female and male participants in terms of (i) the variation in the number of visits, (ii) before and after 5 years of time-lapse after MCI-V, (iii) characterizing difference in visits. Our experiments demonstrate the increased uncertainty in predicting the cognitive status among females as compared to male participants, especially when the visits with significant changes in visits after 5 years of MCI-V.

Leveraging computational intelligence techniques and advanced data analysis methods enables us to delve deeper into the underlying mechanisms and relationships that drive the disease. However, there is a limitation of our study, primarily the small sample size because this study focused exclusively on individuals who developed MCI and subsequently progressed to dementia, excluding those with MCI at the baseline. Although, recent studies in healthcare informatics involving transitions from MCI to Dementia are also observed with small sample sizes [14, 18, 19, 23, 25, 39]. Future studies should aim to include a larger and more diverse cohort, encompassing those who remain at the MCI stage and exhibiting normal cognitive function. This broader scope would allow for a more comprehensive characterization of observed differences and a better understanding of the disease continuum.

While analyzing the variables associated with the MCI and dementia, it is important to distinguish variables that are causally linked to, intrinsic to, or manifest as accompanying symptoms of MCI and dementia. We observed the potentially causally linked variables through our network analyses, such as diabetes and cardiovascular conditions, contributing to the dementia, supporting existing studies [20, 24]. Intrinsic variables, such as Clinical Dementia Rating and scores from the Functional Activities Questionnaire, measuring the direct impact on patient health and daily functioning, providing essential insights into its primary characteristics and severity of cognitive decline as discussed in the previous work [15, 26]. Accompanying symptoms such as neuropsychiatric manifestations affect the quality of life, significantly influencing the patient outcomes. We observed the difference in the accompanying symptoms with the minor changes in Euclidean distance in Fig. 7(c) where men and women have little changes in accompanying symptoms ranging from 0 to 3 as compared to the scores changed with infusion of intrinsic variables with Euclidean distance of up to 35.

## Conclusion

Network analysis provides valuable insights associated with the onset of dementia by characterizing its progression from initial MCI diagnosis. The assortative nature of the graph formed through correlations between variables allows better understanding of variable relationships as MCI progresses to dementia. Our findings have elucidated the dynamic nature of these relationships, identifying key variables that contribute to the onset of dementia. Additionally, our investigation has revealed potential differences in the effects of these variables based on sex, suggesting the need for sex-specific models in the future. Further research is encouraged to expand upon these findings, encompassing larger and more diverse populations to enhance the generalizability of the results. By continuing to investigate the intricate associations among variables, we can advance our understanding of dementia and strive to improve prevention, management, and care for affected individuals and their families.

## Data Availability

The MCSA study makes de-identified data available to qualified researchers upon reasonable request. The programming script is publicly available at Github: https://github.com/drmuskangarg/MCI2DEM.

## Abbreviations

CDR: Clinical Dementia Rating
DEM-V: Dementia visit
M2D-V: Visit between Mild Cognitive Impairment and Dementia
MCI: Mild Cognitive Impairment
MCI-V: First Mild Cognitive Impairment visit
MCSA: Mayo Clinic Study of Ageing
